# An Unusual Case of Intrapulmonary Placement of a Thoracic Drain

**DOI:** 10.7759/cureus.99867

**Published:** 2025-12-22

**Authors:** Akram Assadiki, Hamraoui Salima, Doaae El Ouaddane, Ahdadi Ahmed, Rachid Marouf

**Affiliations:** 1 Department of Thoracic Surgery, Faculty of Medicine and Pharmacy, Mohammed VI University Hospital, Mohammed First University, Oujda, MAR; 2 Department of Thoracic and Cardiovascular Surgery, Faculty of Medicine and Pharmacy, Mohammed VI University Hospital, Mohammed First University, Oujda, MAR

**Keywords:** chest tube, iatrogenic, lung perforation, spontaneous pneumothorax, video-assisted thoracoscopic surgery (vats)

## Abstract

Chest tube insertion for pneumothorax and other pleural diseases is a common and relatively minor procedure but carries risks such as iatrogenic lung injury, particularly in patients with preexisting lung disease or pleural adhesions. We present a case of lung injury following chest tube insertion in a Moroccan man with chronic obstructive pulmonary disease (COPD) and emphysematous lung disease. The chest tube traversed lung tissue, causing a persistent pneumothorax and subcutaneous emphysema. Surgical intervention, including mini thoracotomy, adhesiolysis, and lung resection, was required. The patient recovered satisfactorily. This report highlights the importance of correct technique, imaging for accurate tube placement, and timely surgical intervention in complicated cases. Although rare, lung injury caused by chest tube insertion is serious and warrants careful attention from trained caregivers.

## Introduction

The placement of a chest tube is a common technique adopted in the management of hemothorax, empyema, and pneumothorax. Even though technically simple, chest tube placement has a significant risk of complications, of which iatrogenic lung injury has been reported in approximately 3% of patient cases [1]. Such a situation is more probable when pleural adhesions and underlying lung disease are involved [2]. This intraparenchymal injury may lead to a bronchopleural fistula, a rare but documented complication [3]. These complications most typically occur during inexperienced physician procedures, although even skilled practitioners are vulnerable, and management ranges from conservative measures to surgical intervention, including anatomical lung resection [4].

## Case presentation

**Figure 1:**
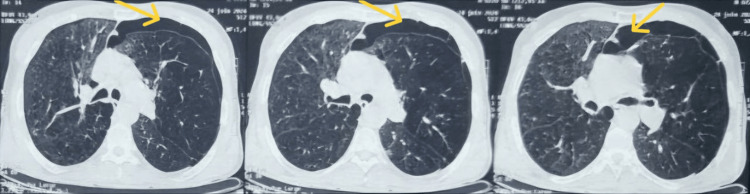
Computed tomography before chest tube insertion The admission chest computed tomography scan revealed an anterior left-sided pneumothorax (indicated by yellow arrows)

A 56-year-old Moroccan man, a chronic smoker with a history of chronic obstructive pulmonary disease (COPD) and emphysematous lungs, presented with shortness of breath and chest pain. He was diagnosed with a left-sided secondary pneumothorax. On admission, his vital signs were stable: heart rate of 80 beats per minute (bpm), blood pressure of 120/71 mmHg, and oxygen saturation of 93% while receiving 8 L/minute of oxygen. He showed signs of respiratory distress with paradoxical thoracoabdominal movement, and clinical examination revealed a left-sided air effusion syndrome. A chest X-ray, followed by computed tomography (CT), confirmed the left pneumothorax (Figure 1). 

**Figure 2:**
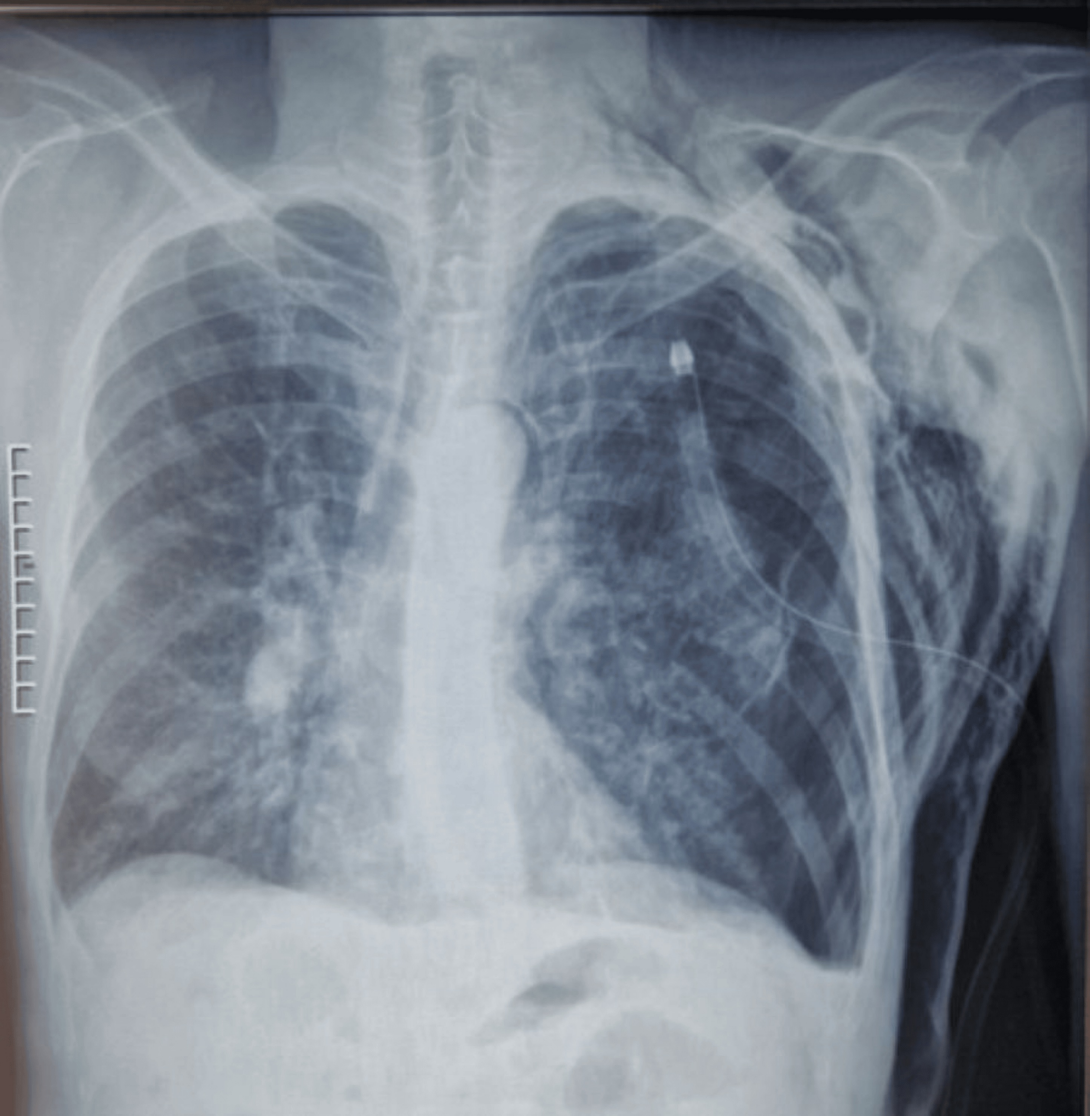
Chest X-ray taken one hour after chest tube insertion The chest X-ray shows a persistent pneumothorax associated with significant subcutaneous emphysema

The patient was admitted to pulmonology, and a 24 Fr Jolly (Vygon, Swindon, United Kingdom) chest tube was placed in the fourth intercostal space. A follow-up chest X-ray showed persistent pneumothorax with extensive subcutaneous emphysema (Figure 2), and air leaks continued.

**Figure 3:**
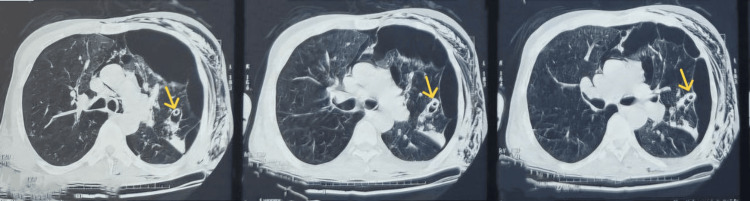
Computed tomography performed a day after chest tube insertion The scan shows the chest tube positioned in the left upper lobe (indicated by yellow arrows), with lung collapse appearing more severe than prior to the insertion

A computed tomography performed the next day revealed the chest tube tip lodged within the upper lobe of the left lung, creating a parenchymal-pleural fistula and worsening subcutaneous emphysema (Figure 3).

As drainage was ineffective in controlling the air leak, the patient was transferred to thoracic surgery.

**Figure 4:**
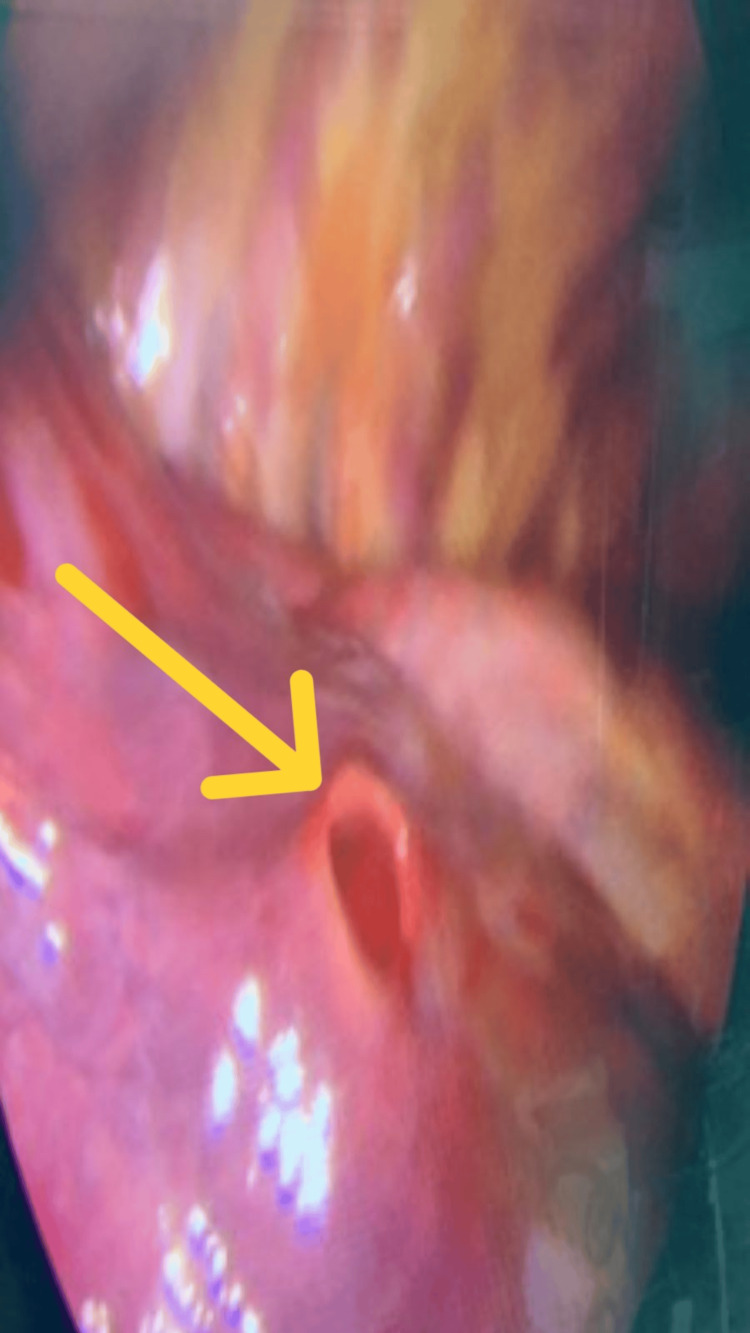
Intraoperative image The wound tract was readily identified through thoracoscopic exploration (indicated by the yellow arrow)

The following day, video-assisted thoracoscopic surgery (VATS) was performed via the fifth intercostal space, with an additional 2 cm camera port in the seventh intercostal space. Thoracoscopic exploration (Figure 4) identified the intrapulmonary drain tract, with entry at the drainage port site and exit at the apex. Adhesions between the chest wall and left upper lobe were dissected using Harmonic scissors (Ethicon, Raritan, NJ). Apical lung resection was completed with four Echelon (Ethicon, Raritan, NJ) stapler cartridges, and an additional parenchymal resection was performed at the inlet port with two cartridges. Hemostasis was secured; two chest tubes, one apical and one basal, were placed, and the wound was closed in layers.

**Figure 5:**
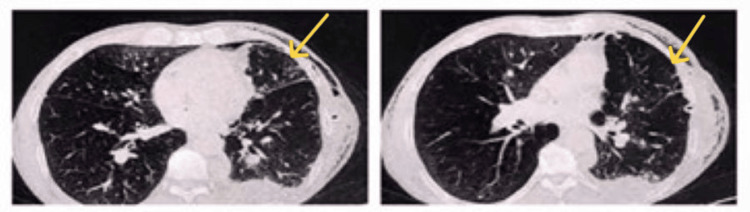
A follow-up thoracic computed tomography Follow-up chest computed tomography obtained on postoperative day 5 shows the appropriate re-expansion of the lung, with the left lung adequately opposed to the thoracic wall (yellow arrows)

**Figure 6:**
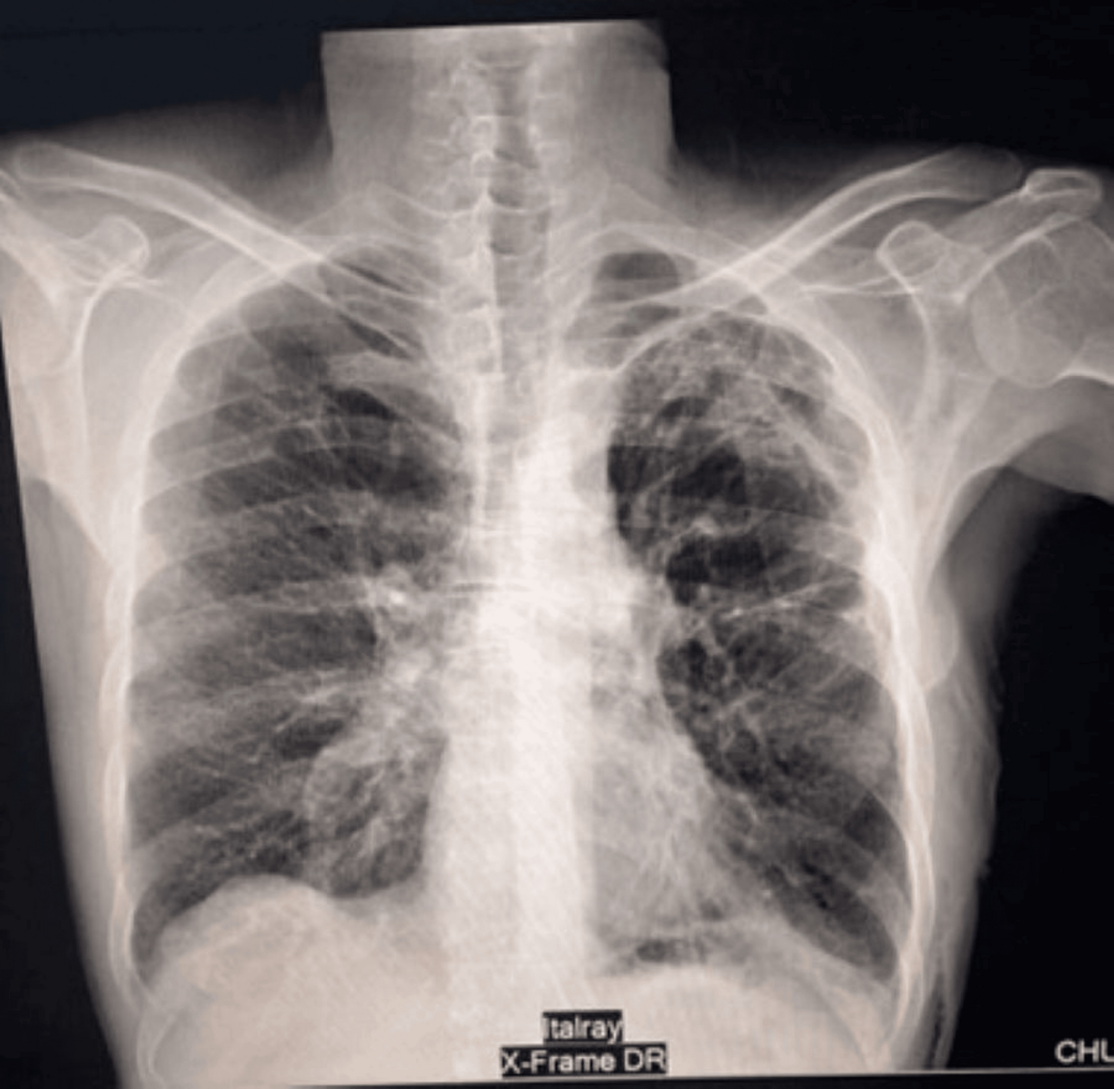
The discharge chest X-ray The patient's discharge chest X-ray was satisfactory

Postoperatively, no further air leakage was observed, and the lung expanded fully. The recovery was uneventful. The patient was discharged in good condition following chest tube removal, with satisfactory control computed tomography imaging (Figure 5) and a proper follow-up chest X-ray (Figure 6).

## Discussion

Chest tube thoracostomy remains a vital intervention in critically injured patients, as it allows for the evaluation of intrathoracic bleeding, facilitates the evacuation of pleural blood, prevents the development of tension pneumothorax, and promotes lung re-expansion; in addition, it contributes to the stabilization of low-pressure pulmonary vessels and enhances overall respiratory function [5]. Reported complications include insertional (7.9%), positional (11.8%), and infectious (2.6%), with overall complication rates ranging from 1.2% to 15% [6,7]. Although complications are more frequent among trainees, they may occasionally occur even with experienced specialists.

The most frequent complications are bleeding from intercostal vessel injury, lung parenchymal damage, and persistent air leaks. Rare but serious events have also been described, including accidental placement of the tube into the diaphragm or the phrenic nerve [8]. In exceptional cases, injuries to major structures such as the aorta, pulmonary arteries, cardiac chambers, or even the stomach and abdominal cavity through the diaphragm have been reported [9].

Multiple factors contribute to technical difficulties, particularly when chest tubes are inserted by junior physicians without close supervision. The site of entry plays a critical role: the safest location is usually the fifth intercostal space in the midaxillary line, where the intercostal space is widest and away from vital structures. Positioning the patient semi-recumbent or upright improves landmark identification and reduces the risk of misplacement into breast tissue. Although trocar tubes allow easier insertion, they must be carefully controlled to avoid injury, especially in patients with adhesions.

The blind insertion of a chest tube in cases of loculated empyema or areas with pleural adhesions should be avoided, as it often leads to ineffective drainage and increased risk of complications. Furthermore, the misdiagnosis of conditions such as bullae, lung abscesses, or diaphragmatic eventration carries the risk of improper chest tube placement, including possible insertion into the abdominal cavity [10].

Lung perforation remains a recognized complication, though uncommon in adults due to blunt dissection. Such injuries are more likely in patients with reduced lung compliance or dense pleural adhesions and may evolve into persistent parenchymal-pleural fistulas. Clinically, a malpositioned intrapulmonary tube often presents with subcutaneous emphysema and crepitus from persistent air leaks, though some patients may remain asymptomatic and without radiographic evidence. Ideally, tubes for pneumothorax should be placed anteriorly and superiorly. On chest radiographs, they typically follow a smooth curvilinear path into the rib cage, convex laterally if posterior and medially if anterior. CT imaging offers the most accurate assessment, often revealing intraparenchymal tube placement, associated hematoma, residual pneumothorax, and subcutaneous emphysema. Surgical repair is rarely necessary, except in cases of uncontrolled air leaks or major hemorrhage.

## Conclusions

Chest tube-related lung parenchymal injury is rare, with limited guidance available for its management. While many cases resolve with conservative treatment, patients with preexisting lung disease may require surgery. This case highlights the successful surgical management of an inadvertent intrapulmonary chest tube placement and underlines the need for thoracic surgeons to be prepared for such uncommon but serious complications.

## References

[1] Ball CG, Lord J, Laupland KB (2007). Chest tube complications: how well are we training our residents?. Can J Surg.

[2] Filosso PL, Guerrera F, Sandri A, Roffinella M, Solidoro P, Ruffini E, Oliaro A (2017). Errors and complications in chest tube placement. Thorac Surg Clin.

[3] Paramasivam E, Bodenham A (2008). Air leaks, pneumothorax, and chest drains. Cont Educ Anaesth Crit Care Pain.

[4] Haron H, Rashid NA, Dimon MZ, Azmi MH, Sumin JO, Zabir AF, Abdul Rahman MR (2010). Chest tube injury to left ventricle: complication or negligence?. Ann Thorac Surg.

[5] Daly RC, Mucha P, Pairolero PC, Farnell MB (1985). The risk of percutaneous chest tube thoracostomy for blunt thoracic trauma. Ann Emerg Med.

[6] Seiden SC, Barach P (2006). Wrong-side/wrong-site, wrong-procedure, and wrong-patient adverse events: are they preventable?. Arch Surg.

[7] Bailey RC (2000). Complications of tube thoracostomy in trauma. J Accid Emerg Med.

[8] Nahum E, Ben-Ari J, Schonfeld T, Horev G (2001). Acute diaphragmatic paralysis caused by chest-tube trauma to phrenic nerve. Pediatr Radiol.

[9] Içöz G, Kara E, Ilkgül O, Yetgin S, Tunçyürek P, Korkut MA (2003). Perforation of the stomach due to chest tube complication in a patient with iatrogenic diaphragmatic rupture. Acta Chir Belg.

[10] Etoch SW, Bar-Natan MF, Miller FB, Richardson JD (1995). Tube thoracostomy. Factors related to complications. Arch Surg.

